# Alternating Dynamics of *oriC*, SMC, and MksBEF in Segregation of Pseudomonas aeruginosa Chromosome

**DOI:** 10.1128/mSphere.00238-20

**Published:** 2020-09-09

**Authors:** Hang Zhao, Bijit K. Bhowmik, Zoya M. Petrushenko, Valentin V. Rybenkov

**Affiliations:** a Department of Chemistry and Biochemistry, University of Oklahoma, Norman, Oklahoma, USA; University of Wyoming

**Keywords:** condensins, MksBEF, SMC, chromosome structure, *Pseudomonas aeruginosa*, MksB, SMC proteins

## Abstract

Condensins are essential for global chromosome organization in diverse bacteria. Atypically, the Pseudomonas aeruginosa chromosome encodes condensins from two superfamilies, SMC-ScpAB and MksBEF. Here, we report that the two proteins play specialized roles in chromosome packing and segregation and are synthetically lethal with ParB. Inactivation of SMC or MksB affected, in a protein-dependent manner, global chromosome layout and its timing of segregation and sometimes triggered a chromosomal inversion. The localization pattern was also unique to each protein. SMC clusters colocalized with *oriC* throughout the cell cycle except shortly after origin duplication, whereas MksB clusters emerged at cell quarters shortly prior to *oriC* duplication and stayed there even after cell division. The relocation of the proteins was abrupt and coordinated with *oriC* dynamic. These data reveal that the two condensins play distinct dual roles in chromosome maintenance by organizing it and mediating its segregation. Furthermore, the choreography of condensins and *oriC* relocations suggest an elegant mechanism for the birth and maturation of chromosomes.

**IMPORTANCE** Mechanisms that define the chromosome as a structural entity remain unknown. Key elements in this process are condensins, which globally organize chromosomes and contribute to their segregation. This study characterized condensin and chromosome dynamics in Pseudomonas aeruginosa, which harbors condensins from two major protein superfamilies, SMC and MksBEF. The study revealed that both proteins play a dual role in chromosome maintenance by spatially organizing the chromosomes and guiding their segregation but can substitute for each other in some activities. The timing of chromosome, SMC, and MksBEF relocation was highly ordered and interdependent, revealing causative relationships in the process. Moreover, MksBEF produced clusters at the site of chromosome replication that survived cell division and remained in place until replication was complete. Overall, these data delineate the functions of condensins from the SMC and MksBEF superfamilies, reveal the existence of a chromosome organizing center, and suggest a mechanism that might explain the biogenesis of chromosomes.

## Abstract (precis)

Mechanisms that define the chromosome as a structural entity remain unknown. Key elements in this process are condensins, which globally organize chromosomes and contribute to their segregation. This study characterized condensin and chromosome dynamics in Pseudomonas aeruginosa, which harbors condensins from two major protein superfamilies, SMC and MksBEF. The study revealed that both proteins play a dual role in chromosome maintenance by spatially organizing the chromosomes and guiding their segregation but can substitute for each other in some activities. The timing of chromosome, SMC, and MksBEF relocation was highly ordered and interdependent, revealing causative relationships in the process. Moreover, MksBEF produced clusters at the site of chromosome replication that survived cell division and remained in place until replication was complete. Overall, these data delineate the functions of condensins from the SMC and MksBEF superfamilies, reveal the existence of a chromosome organizing center, and suggest a mechanism that might explain the biogenesis of chromosomes.

## INTRODUCTION

Pseudomonas aeruginosa is a significant opportunistic human pathogen responsible for a variety of infectious diseases in immunocompromised patients and those with a breached cutaneous barrier (1, 2). The high pathogenicity of P. aeruginosa resides in its numerous encoded virulence factors, a cell envelope that is virtually impermeable to most available antibiotics, a plethora of metabolic enzymes suitable for diverse niches, and a sophisticated signaling network that accelerates adaptation of the bacterium to hostile environment (3–6). Recent studies revealed that the global chromosome dynamics in P. aeruginosa are also under an elaborate control that is integrated into the epigenetic and virulent behavior of the bacterium (7).

Key elements in this system are condensins, the characteristically V-shaped ABC-type ATPases that form macromolecular clamps on DNA that can bridge distant DNA fragments and have an intrinsic ability to assemble into a macromolecular scaffold at the core of chromosomes (reviewed in references 8–10). In bacteria, these proteins, besides binding elsewhere on DNA, form dynamic clusters in the middle of nucleoids, where they presumably act as chromosome organizing centers (11–14). Condensins act in cooperation with ubiquitous other nucleoid-associated proteins to impose a multilayer organization of the bacterial chromosome (15, 16).

Three families of condensins have been identified in bacteria. The oldest known condensin, MukBEF, is found in Escherichia coli and related Gammaproteobacteria (17). Most other bacteria carry the SMC-ScpAB complex (18). Its SMC subunit is highly homologous to the archaeal and eukaryotic SMC proteins, but not MukB. Members of the third family, MksBEF, sporadically occur in diverse bacteria, including many environmental and pathogenic strains, typically in combination with SMC-ScpAB or MukBEF (19). Several families of MksBEF were discovered, each with a distant homology to another and some traceable to the E. coli MukBEF. In this respect, MukBEF and MksBEF can be viewed as a large and diverse superfamily of condensins.

Despite sequence divergence and a number of biochemical distinctions, MukBEF and SMC appear to play similar roles in their host bacteria. Both proteins are able to bridge DNAs, form clusters at the core of the chromosome that relocate within the cell in parallel with duplicating chromosomes, and are essential for faithful chromosome segregation (9, 20, 21). However, their DNA recruitment mechanism appears idiosyncratic to the protein. SMC proteins are loaded onto the chromosome in the vicinity of the origin of replication, *oriC*, with the help of the ParAB*S* system (22, 23), whereas MukBEF employs DNA topoisomerase IV and MatP for its recruitment to the *ori* and *ter* domains of E. coli, respectively (24–26).

To highlight the differences between the two condensins, we explored chromosome segregation in P. aeruginosa, which encodes both SMC and MksBEF and in which the defects of condensin inactivation are reasonably well tolerated. The former factor helps reveal the differences in protein localization, if any, whereas the latter reduces the secondary effects of chromosome disorganization caused by condensin inactivation. These benefits of the system allow one to address a cornerstone question in condensin biology, namely, whether the proteins provide the driving force for chromosome segregation or simply follow the segregating chromosomes.

The chromosome of P. aeruginosa is longitudinally organized, with the origin of replication, *oriC*, located at 80% of the cell length and the replication terminal region at the opposite, newly formed pole of the cell. Replication occurs in the middle of the cell, where the replisome remains for the duration of the cell cycle (27, 28). Segregation begins at the *oriC* locus and proceeds bidirectionally along the two chromosomal arms. Unlike E. coli, P. aeruginosa lacks the *ter*-Tus system (8). Instead, segregation ends in the vicinity of *dif*, the site of the chromosome dimer resolution. This arrangement is maintained even in the asymmetric chromosome of P. aeruginosa strain PAO1-UW, in which the two chromosomal arms differ by 60%. In contrast, replication proceeds at the same rate along the two arms (28).

We show here that this pattern of segregation changes upon deletion of condensins in a protein-dependent manner and that the effect differs between the *oriC* region and the rest of the chromosome. In particular, the bulk chromosome segregation was delayed in MksB-deficient cells but occurred sooner in an *smc* mutant. For *oriC*, however, the deletion of any of the condensins produced the same effect, a delay in segregation of one of the sister origins of replication. The deletion of *smc* or *mksB* reorganized or dispersed the large domains previously found in the longer arm of the PAO1-UW chromosome. Moreover, MksB-deficient cells underwent a large chromosomal inversion, which equalized the length of its arms and realigned *dif* and the terminus of replication. In *smc* mutants, segregation proceeded symmetrically and terminated opposite from *oriC* rather than at *dif*. Thus, SMC and MksB have distinct functions in global chromosome folding and segregation. Accordingly, MksB and SMC clusters rarely colocalized with each other. While SMC was primarily found at *oriC*, MksB was located at the midcell for most of the cell cycle. Relocation of both proteins was tied to *oriC* segregation, which is suggestive of a cause and effect relationship in the process. Overall, these data suggest that condensins comprise the core of a chromosome-organizing center in P. aeruginosa that begins its life at a newly formed origin of replication and later matures into an assembly in the middle of the cell.

## RESULTS

### SMC and MksBEF form separate clusters within the chromosome.

**FIG 1:**
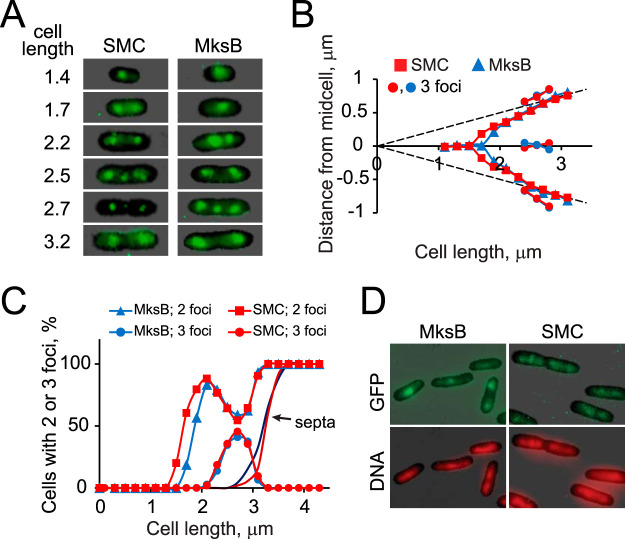
Subcellular localization of MksB and SMC in rapidly growing P. aeruginosa cells. (A) Focal localization of green fluorescent protein (GFP)-tagged MksB and SMC. Cell length is measured in μm. (B). Average position of MksB and SMC foci in cells (*n *> 500; random cell orientation) with two or three foci. Localization of the foci was quantified using the Nucleus program (46). (C) Predominance of cells with two or three foci. Also shown are the proportions of cells containing a septum. Note that the formation of the three-focus cells parallels a decline in two-focus cells (D). Colocalization of MksB-GFP and SMC-GFP with DNA.

We first examined subcellular localization of green fluorescent protein (GFP)-tagged MksB and SMC in P. aeruginosa cells grown in rich medium. Both proteins formed distinct clusters in the middle of short cells and at about quarter positions of longer cells (Fig. 1A). Such localization is characteristic of bacterial condensins (11–13) and is also reminiscent of *oriC*-proximal regions of the P. aeruginosa chromosome (27). Both MksB and SMC were found in the middle of nucleoids (Fig. 1D), which is further consistent with their expected role in chromosome organization. Notably, MksB foci duplicated later than SMC foci (Fig. 1B and C), indicating that they might associate with different intracellular targets and might be playing distinct roles.

Shortly before septum constriction, a third focus could be observed in the middle of the cell. This focus formed only transiently, after the split of the main foci, and soon dispersed at the onset of septum constriction (Fig. 1C). MksB and SMC produced the third focus simultaneously, suggesting the same mechanism for its formation. In all cases, the third focus was found in the middle of a DNA mass and not at a periphery of the nucleoids (Fig. 1D). Moreover, its location in the middle of the cell suggested other attractors than duplicating *oriC* sites in the daughter cells. Piqued by these unusual features, we further explored chromosome segregation in P. aeruginosa.

### Deletion of condensins desynchronizes segregation of *oriC*.

**FIG 2:**
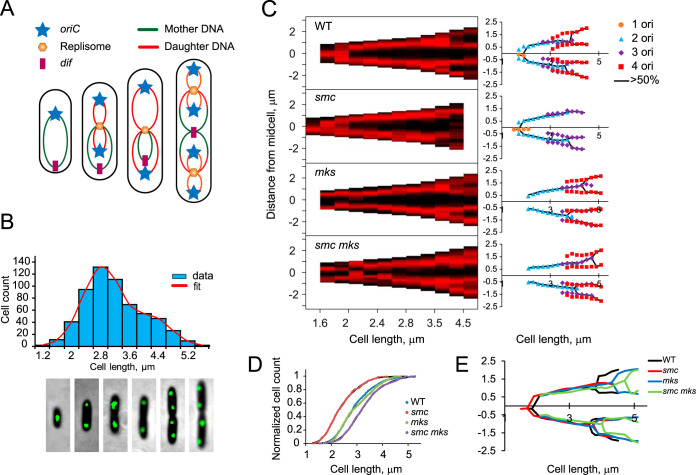
Segregation of the origin of replication in condensin-deficient cells. (A) Layout of the P. aeruginosa chromosome during replication. (B) Location of *oriC* in representative cells (bottom) matched with the bimodal distribution of cell sizes (top). (C) Binned demographs of condensin variants (left, *n *> 300) and average position of *oriC* as a function of cell length (right). On demographs, the cell outline is shown in black and the intensity of *oriC* fluorescence in red. Cells were rotated to score single *oriC* foci and two foci in the three-focus cells at negative coordinates. The black lines on the average graphs trace the position of *oriC* in the dominant (>50%) population of cells. (D) Cumulative cell size distribution in wild-type and condensin-deficient cells. Data were fitted to an integrated double Gaussian distribution. The doubling times for wild-type, *smc*, *mksB*, and *smc mksB* cells were, respectively, 55, 79, 51, and 59 min. (E) Comparison of *oriC* segregation in condensin-proficient and -deficient cells.

Subsequent experiments were carried out with cells grown in minimal medium, with replication initiated only once per cell cycle. The *oriC*-proximal region was tagged with *tetO* repeats and visualized using a fluorescent repressor operator system (FROS) as previously described (27, 28). Its location was followed in parental PAO1-UW cells and in cells deficient in one or both condensins. As previously shown (27, 28), the chromosome in P. aeruginosa is stretched along the major axis of the cell from *oriC* to *dif*. At the onset of replication, *oriC* moves to the midcell, duplicates, the daughter *oriC* sites then move apart, and the mother chromosome is gradually pulled toward the replisome, which is located in the middle of the cell (Fig. 2A).

In agreement with previous studies, most of the newly born P. aeruginosa cells contained a single *oriC* (Fig. 2B and C). When cells grew up to 1.6 μm, *oriC* relocated to the middle of the cell. Duplication of *oriC* occurred at a cell length of 1.8 μm, after which sister *oriC* sites moved apart and then gradually migrated toward the 20%/80% positions. At cell length of 3.6 μm, the second round of *oriC* duplication could be observed. Cell division did not correlate with *oriC* duplication and could occur before or after it, giving birth to shorter cells with a single *oriC* or longer ones with two origins of replication (see Fig. S1A in the supplemental material). A delay in cell division relative to the second round of chromosome replication probably explains the bimodal distribution of cell lengths (Fig. 2B).

10.1128/mSphere.00238-20.1 FIG S1 Typical *oriC* segregation patterns in *oriC*-tagged PAO1 (A) and SMC-deficient (B) strains. Early exponential-phase cells (optical density at 600 nm [OD_600_] of 0.1) were observed using time-lapse microscopy at 5-min intervals between frames. (A) PAO1 *tetO-*PA0069 cells. All cells that underwent division during the time series were scored and classified according to the number of *oriC* foci in daughter cells. Shown are the number of cells in each category and representative examples. (B) *smc tetO-*PA0069 cells. All cells longer than 3 μm were counted and classified. The average length (plus or minus standard deviation [SD]) of scored cells is shown below the images. Download FIG S1, PDF file, 0.6 MB. Copyright © 2020 Zhao et al. 2020 Zhao et al. This content is distributed under the terms of the Creative Commons Attribution 4.0 International license.

Deletion of *smc* altered the aforementioned pattern in two ways. First, the average cell length declined to 2.3 μm, compared to the 2.8 μm found in wild-type cells (Fig. 2D). Second, segregation often happened only in one of the sister *oriC* sites prior to cell division (70%), resulting in a large number of newborn cells with a single origin of replication (Fig. 2C, second panel). In addition, cell division also frequently occurred in two-focus cells (Fig. S1B). Of note, cell division proceeded slower in three- than four-focus cells, resulting in a lower percentage of completed events during observation using time-resolved microscopy (Fig. S1B).

Deletion of *mksB* had an opposite effect on cell morphology. Although the average cell length was similar to that of the parental strain (Fig. 2D), the cell division event was delayed relative to the second round of origin duplication (Fig. 2C, third panel). As a result, virtually no newborn cells with a single *oriC* could be found. Moreover, we observed a pronounced delay in separation of the two sister *oriC* sites. Although not entirely synchronous, the two *oriC* sites in wild-type cells separated over a narrow range of cell lengths, averaging 3.6 and 3.8 μm for the first and second events, respectively. In *mksB* cells, the lengths were 3.9 and 4.5 μm, respectively.

Remarkably, the deletion of both condensins did not produce dramatic effects on *oriC* segregation. The morphology of *smc mksB* cells was similar to that of the *mksB* mutant, except that the delay in segregation of *oriC* sites became more pronounced (Fig. 2C and E) and the average cell length increased to 3.2 μm (Fig. 2D). Thus, neither of the condensins is required for segregation of *oriC* sites, alone or together; however, both are needed to ensure the synchronicity of *oriC* segregation in the two daughter cells.

### A delay between replication and segregation of *oriC* sites.

**FIG 3:**
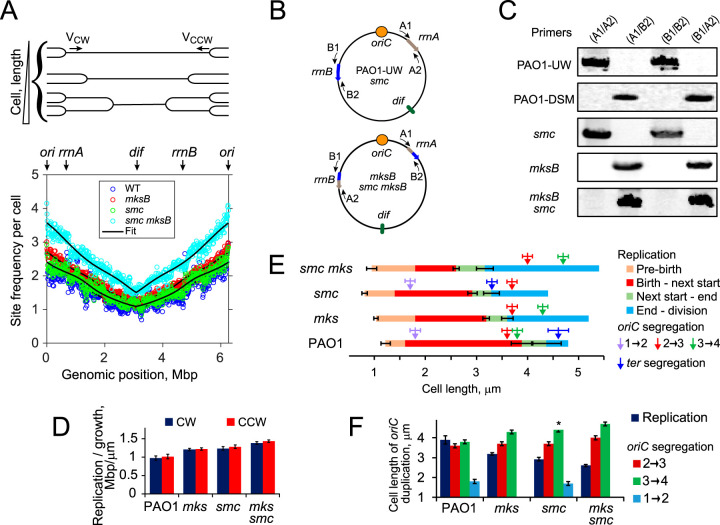
Chromosome replication in the presence and absence of condensins. (A) Marker frequency analysis of chromosome replication. Cell culture contains multiple cell populations where bidirectional DNA replication advanced to its own extent, resulting in a higher copy number of origin-proximal regions (top). Bottom panel shows frequencies of all detected chromosomal sites for the four condensin variant strains fitted to the model that postulates a constant rate of replication. For *mksB* and *smc mksB* strains, the genomic position takes into account the inversion between *rrnA* and *rrnB*. (B, C) PCR analysis of the genomic connectivity at *rrnA* and *rrnB* sites. Primers A1, A2, B1, and B2 are homologous to genomic regions flanking the rRNA gene sites (B). Both MksB-deficient strains carry the same inversion as that found in PAO1-DSM (C). (D) Best fit relative (i.e., normalized to the cell growth rate) chromosome replication rates for the clockwise (CW) and counterclockwise (CCW) forks for the parental strain and condensin mutants. Note that the copy number of all sites is greater in *mksB* and especially in *smc mksB* mutants than in wild-type cells due to the higher frequency of long cells and the greater extent of replication in these cultures (Fig. 2D). (E) Midpoints of *oriC* segregation events from Fig. 2E and the replication terminus (*ter*) from Fig. 4F mapped onto the cell cycle of the four condensin variant strains. In all cases, replication is predicted to start prior to the birth of the cell. No *ter* segregation was observed in *mksB* cells. Error bars show standard error of the mean (SEM) for fitting data to the model. (F) The best fit cell length of *oriC* replication in condensin mutants compared to the cell length at which *oriC* sites are segregated in the two daughter cells. The segregation data are as determined in Fig. 2E for the transitions from two- to three-*oriC* cells (2→3), from three- to four-*oriC* cells (3→4) and, when observed, from one- to two-*oriC* (3→4) cells. For *smc* mutants, the second *oriC* duplication was rarely observed, and the bar represents the low bound on the expected cell length at the duplication (*).

The delay found in chromosome segregation could in principle be caused by a delay in replication. To evaluate this possibility, we explored chromosome segregation in condensin mutants using marker frequency analysis. To this end, chromosomal DNA of the mutant cells was subjected to deep sequencing, and the relative abundances of genomic loci fit to a model that postulates up to two replication start events at the *oriC* during the lifetime of the cell (28). This analysis quantifies duplication of genomic loci following the passage of a replication fork, which leads to a higher copy number of origin-proximal DNA fragments (Fig. 3A).

Unexpectedly, we found discontinuities in the marker frequency patterns of *mksB* and *smc mksB* cells (see Fig. S2 in the supplemental material). PCR analysis of the implicated region revealed that they are caused by a large chromosomal inversion between the *rrnA* and *rrnB* regions, which occurred after the deletion of the *mksB* gene (Fig. 3B and C). This inversion was found in all 25 analyzed Δ*mksB* colonies obtained after 5 separate conjugations, suggesting that it is tightly linked to *mksB*. Notably, the same inversion was reported for the PAO1-DSM lineage of PAO1 strains, where it is accompanied by a deletion in *mksE* (29, 30). Apparently, a fully functional MksBEF is required to maintain the inversion found in PAO1-UW cells. The existence of this inversion is accounted for in further analysis. This inversion was not responsible for the *oriC* segregation defects reported in Fig. 2, since the reintroduction of *mksB* back into Δ*mksB* and Δ*smc* Δ*mksB* cells restored the segregation pattern expected for their parents (see Fig. S3 in the supplemental material) but not the inversion itself.

10.1128/mSphere.00238-20.2 FIG S2 Marker frequency analysis of chromosome replication in condensin-deficient cells without corrections for the chromosomal inversion. Genomic location of the quantified sites is shown using the chromosome of PAO1-UW as a reference. The discontinuities detected in *mksB* and *smc mksB* cells mark the junctions of the inversion. Download FIG S2, PDF file, 0.5 MB. Copyright © 2020 Zhao et al. 2020 Zhao et al. This content is distributed under the terms of the Creative Commons Attribution 4.0 International license.

10.1128/mSphere.00238-20.3 FIG S3 *oriC* segregation patterns in strains whereto *mksB* was reinserted into its endogenous location. The binned demograms and location of *oriC* is shown for Δ*mksB*::*mksB tetO-*PA0069 (A) and Δ*smc* Δ*mksB*::*mksB tetO-*PA0069 (B) cells. Note the large number of single-focus cells and almost simultaneous segregation of the daughter *oriC* sites in Δ*mksB*::*mksB* cells, which is typical for the wild-type PAO1 strain but not the *mksB* mutant (Fig. 2C). Likewise, the large number of short single-focus cells and long three-focus but not four-focus cells is typical for Δ*smc* but not Δ*smc* Δ*mksB* cells (Fig. 2C). Download FIG S3, PDF file, 0.3 MB. Copyright © 2020 Zhao et al. 2020 Zhao et al. This content is distributed under the terms of the Creative Commons Attribution 4.0 International license.

In all three mutants, replication started at *oriC* and proceeded bidirectionally to terminate opposite from *oriC*, indicating that the clockwise and counterclockwise forks advance with the same rate (Fig. 3D). However, the rate of replication varied from one mutant to another. The deletion of *smc* or *mksB* resulted in 20% faster replication relative to cell growth than that for the wild-type cells, whereas the effect was additively higher (40%) for the double mutant (Fig. 3D).

Figure 3E summarizes key cell cycle events for the three condensin mutants and their parental strain and relates them to *oriC* segregation. In all cases, chromosome replication began prior to the birth of cells. However, the duration of this “prebirth” replication period varied and was notably longer for condensin mutants than for the parental strain, which is fully consistent with the faster replication predicted for the mutants (Fig. 3D). Moreover, the timing between *oriC* replication and segregation was also affected. These were better observed late during the cell cycle, when they were not interjected by cell division. In wild-type cells, the best-fit start of *oriC* replication was virtually simultaneous with segregation of the origin in both daughter cells. In contrast, we observed a clear delay in segregation of *oriC* sites following their replication, which became especially pronounced in the double-condensin mutant (Fig. 3E and F). Thus, the delay in *oriC* segregation in one of the sister cells (Fig. 2) cannot be attributed to *oriC* replication.

### Inactivation of SMC and MksB dissolves all domains and disorganizes the *dif* region.

**FIG 4:**
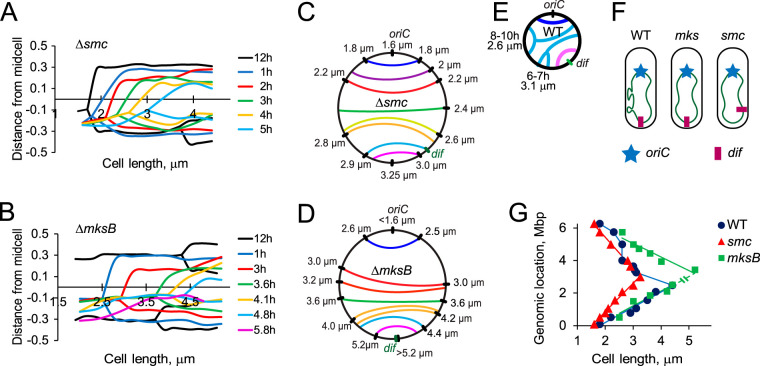
Divergent effects of SMC and MksB inactivation on chromosome segregation. (A, B) Segregation of the tagged chromosomal loci in SMC-deficient (A) or MksB-deficient (B) cells. The intracellular positions of each fluorescently tagged locus are shown as a function of cell length. Cells were rotated to score most of the fluorescent signal at negative distances from the midcell. (C, D) Dial graphs of chromosome segregation which connect sites on the two chromosomal arms that segregate at the same cell length. (E) A dial graph for the parental strain, which was analyzed under identical conditions. Marked are the two large domains on the longer arm together with their location and the cell length at which they segregate. (F) A diagram summarizing chromosome segregation patterns in wild-type and condensin-deficient cells. (G) A summary of chromosome segregation in the three strains. Note that segregation of *dif* was never detected in *mksB* cells.

We next introduced fluorescent tags into various locations of the P. aeruginosa chromosome and examined chromosome segregation in condensin-deficient cells. As reported previously for wild-type P. aeruginosa (28), chromosomal loci of both *smc* and *mksB* mutants migrated toward the middle of the cell, where they duplicated and moved apart (Fig. 4A and B). Focus duplication occurred sequentially in accord with genomic location. The second round of segregation was only detected for the *oriC* region. To facilitate the comparison of all tagged chromosomal sites, we next marked the midpoints of segregation on the map of the chromosome and connected the DNA sites on the opposite arms that segregated at the same cell length (Fig. 4C and D). In the parental PAO1-UW strain, chromosome segregation proceeds sequentially from *oriC* to *dif* along the two chromosomal arms, with the exception of two large domains on the longer arm, which segregate simultaneously (28) (Fig. 4E). No such segregation delays were observed in MksB- or SMC-deficient strains (Fig. 4C and D). Thus, all segregation domains previously observed in PAO1-UW cells were dispersed or reorganized following inactivation of either of the condensins.

Another striking difference between condensin mutants and parental cells was in the site of segregation termination. In wild-type and MksB-deficient cells, the last segregating region was found in the vicinity of *dif* (Fig. 4D). In *mksB* cells, however, owing to the large chromosomal inversion, this region is opposite from *oriC*. This region segregated very late during cell division, and we never found *mksB* cells with two *dif* foci (Fig. 4B, F and G). Apparently, the duplicated *dif* sites remain close to the septum up until the cell division event. This is in contrast to the parental cells, where separation of *dif* foci was routinely observed prior to cell division. We also readily observed segregation of all tagged sites, including *dif*, in SMC-deficient cells (Fig. 4C and G). In this case, however, *dif* was no longer the last segregated site. Thus, inactivation of either SMC or MksB resulted in disorganization of segregation of the *dif* region.

The effect of condensin deletions could also be seen in the rest of the chromosome (Fig. 4G). In MksB-deficient cells, chromosome segregation was markedly delayed compared to that in the wild type, primarily in the left chromosomal arm. In *smc* cells, chromosome segregation happened in shorter cells and in both arms. Notably, these cells were also substantially shorter than the wild-type or MksB-deficient cells (Fig. 2D), revealing effects of the mutation on cell cycle progression. Compared to the cell cycle, the timing of segregation varied depending on mutant and chromosomal location. In particular, *oriC* segregation was delayed compared to its replication in all mutants (Fig. 3E). In contrast, the terminus of replication segregated virtually simultaneously with its replication in *smc* mutants but not in the wild-type cells, and it was delayed even further, almost never observed, in *mksB* mutants.

### Alternating dynamics of *oriC*, SMC, and MksB.

**FIG 5:**
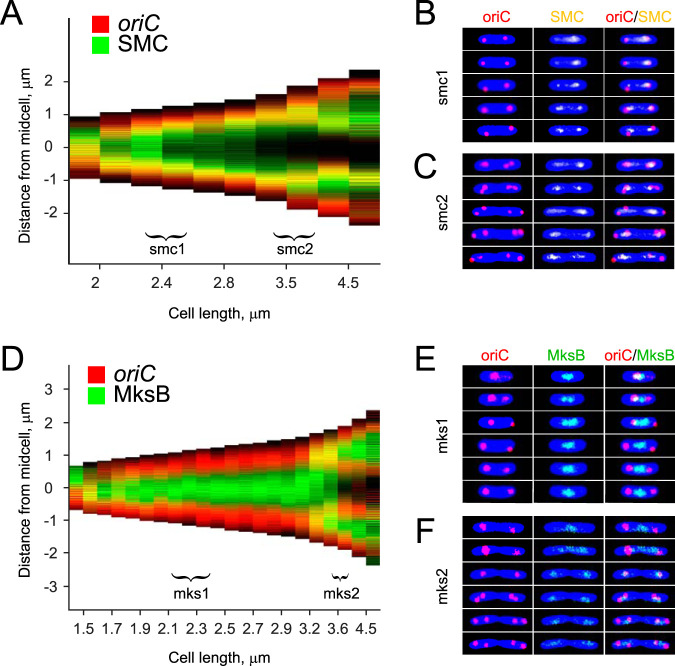
Choreography of *oriC*, SMC, and MksB dynamics. (A) Binned demographs of cells harboring SMC-mVenus and mCherry-tagged *oriC* (*n *= 483). Brackets mark the ranges analyzed in panels B and C. Cell orientation was decided as in Fig. 2. (B and C) Representative time-lapse images collected every 3 min for cells with initial lengths of 2.3 μm (B) and 3.4 μm (C). (D) Binned demographs of cells harboring MksB-GFP and mCherry-tagged *oriC* (*n *= 1,612). (D and E) Representative time-lapse images collected every 3 min for cells with initial lengths of 2.1 μm (E) and 3.5 μm (F).

To further contrast the roles of SMC and MksB in chromosome segregation, we examined localization patterns of the proteins relative to *oriC*. Figure 5A shows binned demographs of cells harboring mCherry-tagged *oriC* and SMC-mVenus. Fluorescence intensity was recorded along the major axis of each cell, cells were binned according to their length, and the intensity was averaged within each bin. The binned intensity profiles were plotted as a function of cell length as images against black projections of cells (Fig. 5A). Representative doubly labeled cells are also shown in Fig. S4 in the supplemental material.

10.1128/mSphere.00238-20.4 FIG S4 Representative cells with mCherry-tagged *oriC* and mVenus-tagged SMC (A) or mClover-tagged MksB (B). Bar, 2 μm. Download FIG S4, PDF file, 1.2 MB. Copyright © 2020 Zhao et al. 2020 Zhao et al. This content is distributed under the terms of the Creative Commons Attribution 4.0 International license.

The shortest cells contained a single *oriC*, which overlapped with a broader single spot of SMC. As the cells grew, *oriC* duplicated, and the two sister *oriC* sites quickly moved apart toward positions at 20% and 80% of the cell length. Most of the SMC-mVenus fluorescence followed one of the *oriC* sites, whereas the other retained only a small fraction of it. Soon thereafter, SMC equalized at the two spots and remained there until the rest of the cell cycle. The sequence of events repeated itself in longer cells, at the next round of *oriC* duplication. The asymmetry of segregation was even clearer in this case. In both daughter chromosomes, the septum-proximal *oriC* retained most of SMC, whereas the poleward origins were slow to recruit the condensin. These data reveal that SMC relocation follows rather than leads relocation of *oriC*. Moreover, the dynamics of SMC appear to be tied to the overall cell morphology.

The dynamics of MksB were strikingly different. MksB and *oriC* did not colocalize in the shortest newborn cells. They did converge onto the midcell as the cells started growing. Once *oriC* duplicated and moved away, however, it also departed from MksB, which remained in the middle of the cell for the duration of the cell cycle. Curiously, the MksB cluster became unstable shortly before the next round of *oriC* duplication. A new MksB spot appeared at one of the *oriC* sites and was soon followed by *oriC* segregation. Segregation of the second *oriC* occurred in a similar manner, with an MksB cluster emerging at the quarter position shortly prior to *oriC* duplication. Other than that, there was no particular order to the events in the sister half-cells, which appeared to occur independent of each other. Once MksB clusters formed at both cell quarters, the one at the midcell faded away. The subsequent cell division completed the cycle by producing daughter cells with a single MksB cluster located at or close to the middle of the cell.

### Condensins are synthetically lethal with ParB.

The prominent roles of SMC and MksB in chromosome organization appear at odds with the modest morphological changes caused by their deletion. To address this apparent incongruity, we examined the genetic interactions of condensins with the ParAB*S* system. We readily constructed strains deficient in ParB and any one of the condensins but could not construct the triple deletion strain. To confirm that this was indeed due to synthetic lethality of ParB, SMC, and MksB, we employed two separate approaches.

**FIG 6:**
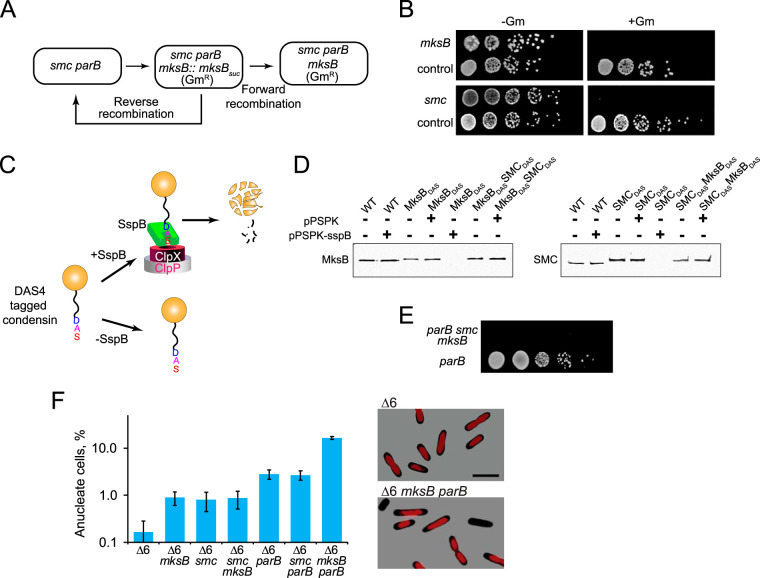
Condensins are synthetically lethal with ParB. (A) Two possible outcomes of a recombinational resolution of a condensin merodiploid strain. (B) Comparative analysis of the forward and reverse recombination in *mksB* (top) or *smc* (bottom) merodiploid strains. The strains were serially diluted and spotted onto LB plates supplemented with 15% sucrose and, when indicated, 15 μg/ml gentamicin. As a control, an unrelated PAO1 strain merodiploid in the *mexGHID* operon, OP578, was used. (C) A diagram of controlled degradation of MksB-DAS4 and SMC-DAS4 proteins upon introduction of an SspB expression plasmid. (D) Immunoblotting analysis of SspB-mediated degradation of DAS4-tagged MksB and SMC in Δ*parB* cells. (E) SspB-mediated degradation of MksB and SMC in Δ*parB* cells is lethal. An SspB expression plasmid was introduced into Δ*parB* Δ*sspB* cells harboring DAS4-tagged MksB and SMC. Cells were then spotted onto LB plates supplemented with gentamicin (30 μg/ml) and IPTG (0.1%). As a control, cells carrying an empty vector were also spotted onto the same plate. (F) Increased frequency of anucleate cells (±SEM) in PΔ6Pore cells deficient for condensins and ParB. Exponentially growing cells (OD_600_ of 0.6) were fixed in 70% ethanol and stained for DNA with 100 nM 4′,6-diamidino-2-phenylindole (DAPI) as previously described (19).

We first constructed a merodiploid Δ*parB* Δ*smc mksB*::*pEX18-sacB-*Δ*mksB-Gm* strain, which contains a functional *mksB*. Plating these cells on sucrose selects for recombinants with an excised plasmid backbone. The excision could proceed forward, completing the deletion of *mksB* and rendering cells resistant to gentamicin (Gm^r^), or backwards, restoring the endogenous *mksB* gene and generating gentamicin-sensitive strains (Fig. 6A). In principle, the probabilities of the two events should be equal, as we found with an unrelated operon MexGHID (Fig. 6B). With MksB, however, all recombination events restored the *mksB-*positive (*mksB^+^*) genotype (Fig. 6B). The same result was observed for a merodiploid Δ*parB* Δ*mksB smc*::*pEX18-sacB-*Δ*smc-Gm* strain when we tried to remove the *smc* gene from Δ*parB* Δ*mksB* cells (Fig. 6B).

To further validate this phenomenon, we employed a bacterial degron system (Fig. 6C), which involves the degradation of specially tagged proteins by ClpXP protease (31, 32). To this end, the gene encoding an adaptor protein for the system, *sspB*, was removed from Δ*parB* cells, and the endogenous *mksB* and *smc* were replaced with their DAS4-tagged versions. In a permissive background, such replacement did not affect the expression levels of the condensins and resulted in a complete degradation of the proteins when a SspB-producing plasmid was introduced into the cells (Fig. 6D). When the plasmid was introduced into Δ*parB smc-DAS4 mksB-DAS4* cells, no colonies could be formed (Fig. 6E). For comparison, transformation of the cells with an empty vector did not impair their growth. We conclude, therefore, that inactivation of SMC, MksB, and ParB is lethal for the cell. However, any one of the three systems is sufficient for cell viability. The found synthetic lethality also offers a straightforward way to verify the functional activity of the two GFP-tagged condensins (see Fig. S5 in the supplemental material).

10.1128/mSphere.00238-20.5 FIG S5 Functional activity of green fluorescent protein (GFP)-tagged SMC and MksB confirmed by generating Δ*parB* Δ*mksB smc*::*smc-gfp* and Δ*parB* Δ*smc mksB*::*mksB-gfp* cells. The *gfp*-tagged versions of condensin genes were used to replace their endogenous copies in Δ*parB* Δ*mksB* or Δ*parB* Δ*smc* cells, as appropriate, and then tested for colony formation. (A and B) Genome organization of the *smc* (A) and *mksB* (B) loci before and after the genes were tagged with *gfp*. Arrows indicate primers used to verify the insertion of *gfp*. (C and D) PCR analysis of the *smc* and *mksB* loci using the primers indicated in panels A and B. (E) Colony formation by the generated mutants and their parental strains. Download FIG S5, PDF file, 0.4 MB. Copyright © 2020 Zhao et al. 2020 Zhao et al. This content is distributed under the terms of the Creative Commons Attribution 4.0 International license.

Given the role of ParB in chromosome segregation, the found synthetic lethality implicates condensins in this function as well. To further substantiate this prediction, we quantified the occurrence of anucleate cells in strains deficient for condensins and ParB. To facilitate uniform DNA staining, we used efflux-deficient PΔ6Pore cells, which are susceptible to numerous drugs (33). In agreement with previous studies (19), the deletion of *mksB* and *smc* resulted in a modest increase in anucleate cell formation (Fig. 6F). Somewhat more anucleate cells, about 3%, were found in *parB* and *parB smc* mutants. This frequency increased dramatically (16% ± 1%) in *parB mksB* cells (Fig. 6F). Thus, all three proteins indeed have functions in P. aeruginosa chromosome segregation.

## DISCUSSION

Uniquely among model bacteria, Pseudomonas aeruginosa encodes two condensins, SMC and MksBEF. At the first glance, this arrangement is redundant. Even though SMC and MksBEF belong to two distinct superfamilies, their activities and functions are close enough to expect the same mechanism, perhaps with species-specific variations. We show here that this is not true. The two proteins have distinct localization patterns, and their deletion produces discernible effects on chromosome structure. At the same time, each of the proteins can compensate for the absence of ParB, while at least one of them must be present to rescue ParB-deficient cells. Hence, MksBEF and SMC asymmetrically perform the same function in chromosome maintenance.

This function is dual in nature. On the one hand, both SMC and MksBEF must be present to stabilize the domain organization of PAO1-UW chromosome (Fig. 4). This feature implicates condensins in stabilization of the global chromosome organization. This conclusion is in accord with both biochemical and genomic studies. The ability of condensins to self-assemble into the chromosome scaffold has been deduced based on *in vitro* reconstitution studies (21, 34). Likewise, a Hi-C analysis of the E. coli chromosome revealed the importance of condensins for a long-range order within the chromosome (15). On the other hand, the ParAB*S* system compensated for the absence of both condensins, indicating that SMC and MksBEF also function in chromosome segregation (Fig. 6). Accordingly, the dynamics of chromosome segregation changed, again in a protein-specific manner, upon inactivation of SMC or MksB (Fig. 2E, 4), which is also consistent with an increased frequency of chromosome missegregation events in condensin mutants (19) (Fig. 6F). Thus, the activity of condensins simultaneously ensures global chromosome organization and segregation.

The fact that SMC and MksBEF perform this function in a distinct manner points to their parallel evolution, namely, that the two proteins evolved separately to accomplish the same task. This conjecture naturally explains why some bacteria carry only SMC or MukBEF/MksBEF. Apparently, a single condensin suffices to ensure faithful chromosome segregation. At the same time, a complete exclusion of condensins from a synthetic genome adversely affected its stability, making condensins an obligatory member of a minimal bacterial genome (35). At the opposite extreme, many pathogenic and environmental bacteria encode multiple condensins. Deletion of condensins from P. aeruginosa was detrimental for its pathogenicity (7). Clearly, having multiple condensins increases the fitness of these bacteria in diverse environment. Conversely, the loss of a condensin might help adapt bacteria to a specialized niche. For example, a spontaneous deletion of *mksE* and *mksF* from P. aeruginosa strain PAO1 created a subline, PAO1-DSM, which also carries a chromosomal inversion (29) and is better adapted for a planktonic growth.

The coexistence of two condensins with partially redundant functions in P. aeruginosa helped reveal aspects of the proteins that were elusive in simpler systems. In particular, it became clear that each of the condensins contributes to the dynamics of *oriC* but in a unique manner. SMC was associated with the *oriC* region throughout the cell cycle except immediately after *oriC* duplication. One of the newly born *oriC* sites was essentially devoid of the condensin and became populated with it only with passage of time. Presumably, *de novo* synthesis of SMC or its recycling from the sister origin is needed to ensure full growth of the SMC cluster. MksB, on the other hand, remained away from *oriC* except shortly before its duplication. It is tempting to conclude from these data that the formation of MksB clusters at *oriC* is causatively linked to its duplication, either directly or indirectly. This could be because *oriC* replication creates a nucleation center for MksB or, vice versa, because relocation of MksB triggers *oriC* duplication. Recently, a mechanism based on the incompatibility of MukB clusters and *oriC* was proposed to explain chromosome segregation in E. coli (36).

Importantly, both SMC and MksB chased *oriC* in its relocation rather than led it. This eliminates the proteins as a plausible driving force for chromosome segregation. Consistent with this was the manner of condensin cluster formation at *oriC*. Both MksB and SMC clusters were highly dynamic and fluctuated widely around their centers. Their formation at new sites occurred abruptly, without clear intermediates or dissolution of clusters at the preceding location. Such a dynamic is characteristic of systems involved in a reaction-diffusion patterning rather than in translocation. It also befits a protein that modulates the activity of another rather than directly dragging it around. Taken together, these data indicate that the role of condensins in chromosome segregation is more passive than active.

The colocalization of SMC with *oriC* is reminiscent of that in other SMC-bearing bacteria, such as B. subtilis, Caulobacter crescentus, and Streptococcus pneumoniae, in which the protein is recruited to the *oriC*-proximal *parS* site in a ParB-dependent manner (22, 37, 38). Parallels can also be found between MksB and MukB. In particular, the MatP-dependent association of MukB with the *ter* region of the E. coli chromosome (24) is not dissimilar to the MksB involvement in bulk chromosome organization. Likewise, the transient colocalization of MksB with *oriC* can be compared to the topoisomerase IV recruitment of MukB to the origin of replication. In this light, it appears that the P. aeruginosa MksB and SMC bear opposite extremes of the same set of activities that their counterparts in single-condensin bacteria learned to perform alone.

An important conclusion from this study is that condensins are not recruited only to the *oriC* region, the middle of the cell, or the cell quarters. They are recruited to all of those locales in a dynamic, growth phase-dependent manner. This was especially clear for MksB, which was transiently recruited to *oriC* concurrent with its duplication and then remained at that site throughout the cell cycle and even past cell division. This dynamic marks cell quarters as a site where MksB clusters are born and the midcell as the place of their dispersal. In this respect, MksB serves as a chromosome organizing center that is born at the time and place of *oriC* duplication and remains there until the mother chromosome completes its replication.

**FIG 7:**
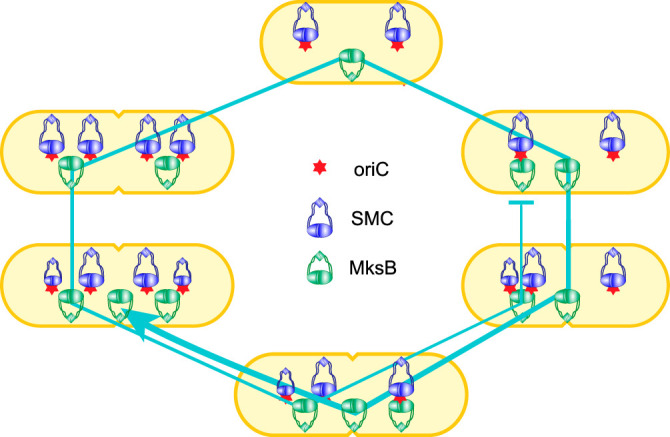
Dynamics of MksB, *oriC*, and SMC during replication and segregation of the P. aeruginosa chromosome (see the text for details). The diagram shows the location of *oriC* and the main clusters of condensins but not that of DNA during cell growth and division. The line traces the life cycle of MksB clusters, which form at the initiation of chromosome duplication and disperse once the duplication is complete and both daughter centers are formed.

Taken together, these data point to an elegant mechanism of chromosome segregation, potentially with ties to cell dynamics (Fig. 7). In this view, the life cycle of the cell has a beginning and an end. The cycle begins with duplication of the origins of replication, which quickly move apart via an as-yet-unknown mechanism. This duplication provides the initial driving force for chromosome segregation. The duplication is triggered by the convergence of MksB onto the site of replication, which marks the first special point inside the cell. In short cells with a single origin of replication, this special point lies in the middle of the cell. Cells with two *oriC* sites have two points of this type, which are located at the quarter positions, and more such points likely emerge in cells with multiple copies of the origin. Further separation of the *oriC* sites is probably driven by entropic forces, which take their roots in the increasing length of replicating chromosomes. Wherever *oriC* sites end up marks the second special point. This second center is populated by SMC for the duration of the cell cycle, until MksB joins it. Thereafter, SMC and *oriC* leave it, and the site matures to become an organizing center for the daughter chromosome. It is quite possible that the daughter clusters coexist with the mother one for a period of time. In PAO1-UW cells in particular, the second round of *oriC* segregation occurs shortly before segregation of the *dif* region, which might explain the transient coexistence of three condensin clusters in one cell. The dispersion of the mother MksB cluster in the middle of the cell, which occurs after the daughters have already been born, marks the demise of the mother chromosome.

Perhaps the most intriguing result of this study is the found link between condensins and the cell architecture. Indeed, SMC was segregating with the septal rather than poleward *oriC* sites during the second round of *oriC* duplication (Fig. 5A). This pattern cannot occur by chance and depends on the SMC access to the information about the structure of the cell. Likewise, the deletion of any condensin resulted in a long delay in *oriC* segregation in one but not the other daughter cell (Fig. 2E). This asymmetry also implies structural differences in the cell caused by inactivation of SMC or MksB. The mechanism of how condensins sense the architecture of the cell may offer clues to the biogenesis of a living cell.

## MATERIALS AND METHODS

### Plasmids and strains.

E. coli strain DH5α (Novagen) was used as a host to construct all the plasmids, whereas E. coli strain SM10 (λ pir) was used for conjugation with P. aeruginosa. P. aeruginosa strains and plasmids used in this study are listed in Tables S1 and S2 in the supplemental material. PAO1 (ATCC 47085) was used as the wild-type strain. Single- and double-condensin deletion mutant PAO1 strains were generated as described previously (7). To tag the chromosomal loci in mutant strains, approximately 500-bp chromosomal segments at the desired locations were amplified using PCR and inserted between HindIII and KpnI restriction sites of the pP30DFRT-*tetO*-0069 plasmid. These plasmids carry approximately 140 *tetO* repeats. These plasmids were inserted into specific locations of the chromosome via homologous recombination.

10.1128/mSphere.00238-20.6 TABLE S1 Strains used in this study. Download Table S1, PDF file, 0.1 MB. Copyright © 2020 Zhao et al. 2020 Zhao et al. This content is distributed under the terms of the Creative Commons Attribution 4.0 International license.

10.1128/mSphere.00238-20.7 TABLE S2 Plasmids used in this study. Download Table S2, PDF file, 0.1 MB. Copyright © 2020 Zhao et al. 2020 Zhao et al. This content is distributed under the terms of the Creative Commons Attribution 4.0 International license.

Plasmid pPSV35Ap-TetR-mCherry was constructed by replacing the TetR-cyan fluorescent protein (CFP) from pPSV35Ap-TetR-CFP with its mCherry-tagged version. This plasmid carries an in-frame fusion of TetR and mCherry genes under the control of the isopropyl-β-d-thiogalactopyranoside (IPTG)-inducible *lacUV5* promoter. Chromosomal tags were visualized by introducing pPSV35Ap-TetR-CFP or pPSV35Ap-TetR-mCherry plasmids into the target cells via electroporation and selecting on LB agar plates supplemented with 200 μg/ml carbenicillin.

To construct pEX18Ap-SMC-mVenus plasmid, approximately 500 bp of the 3′ end of the *smc* gene (PA1527) were linked to a construct encoding peptide HHHHHHHHHGG and *mVenus*. Then, this fused gene, the FLP recombination target (FRT)-flanked gentamicin resistance cassette, and 500 bp downstream of *smc* were inserted into the pEX18Ap plasmid. Similar approach was used to construct pEX18Ap-MksB-GFP and pEX18Ap-SMC-GFP plasmids. Endogenous copies of *mksB* and *smc* were replaced with their fluorescent-tagged versions using allelic exchange method (39). The gentamicin resistance marker (*aac1*) was excised with the help of the pFLP2 plasmid (40). Successful replacements were verified by PCR.

Plasmid pEXG2-ΔParB was constructed by amplifying approximately 500-bp chromosomal segments upstream and downstream of *parB* (PA5562). Resulting fragments were then cloned between HindIII and Pst I restriction sites of the shuttle vector pEXG2 (41). This plasmid was then used to create PAO1 Δ*parB* Δ*smc* (OP435), PAO1 Δ*parB* Δ*mksB* (OP441), PAO1 Δ*sspB* Δ*smc* Δ*parB* (OP475), and PAO1 Δ*sspB* Δ*mksB* Δ*parB* (OP489) strains by allelic exchange. Deletions were subsequently confirmed by PCR. OP498 and OP497 were constructed by replacing the endogenous copies of *smc* and *mksB* in OP130 by their respective DAS4-tagged versions. OP501 and OP508 were constructed by deleting *parB* from OP498 and OP132, respectively. OP505 and OP440 were constructed by deleting *mksB* and *smc* from OP501 and OP508, respectively. OP506 was constructed by replacing the endogenous copy of *mksB* gene by its DAS4-tagged version from OP501. OP529 and OP385 were constructed by introducing chromosomal tags at *oriC*-proximal location following the method described above. The PAO1 Δ*mexGHID* (merodiploid) strain (OP578) was constructed using allelic exchange by mating PAO1 cells with SM10 cells carrying the pEX18Ap-Δ*GHID* plasmid (42). OP579 (Δ*mksB* Δ*parB smc*::*smc-GFP* Gm FRT) and OP580 (Δ*smc* Δ*parB mksB*::*mksB-GFP* Gm FRT) were constructed using allelic exchange by mating OP441 and OP435 with SM10/pEX18AP-*smc*-GFP and SM10/pEX18AP-*mksB*-GFP, as appropriate. The pEX-Δ*mksB*::*mksB* plasmid was constructed by combining the entire *mksB* gene with a 500-bp upstream region amplified from genomic DNA and the FRT-Gm^r^-FRT cassette with the *mksB* downstream region from the pEX18Ap-Δ*mksB* plasmid within the pEX18AP vector. The plasmid was then transferred via conjugation into PAO1 Δ*mksB tetO*-PA0069 using carbenicillin for selection to generate OP581. OP582 was made by allele exchange of PAO1 Δ*mksB* Δ*smc* with pEX*-ΔmksB*::*mksB* followed by the removal of the gentamicin resistance gene using FLP recombinase and transformation of the pP30D-FRT-TetO-0069 plasmid. The PΔ6 strain was constructed by sequential deletion of six efflux pumps from PAO1-UW with the help of plasmids that carried in-frame deletions of appropriate genes. An IPTG-inducible *fhuA* gene was then inserted into this strain using plasmid pGK-LAC-*fhuA* ΔC/ Δ4L (43) to yield PΔ6Pore. Deletions of *parB*, *smc*, and *mksB* in this strain were generated using the appropriate deletion plasmids listed in Table S2.

### Bacterial growth and microscopy.

PAO1 cells harboring chromosomal tags and pPSV35Ap-TetR-CFP or pPSV35Ap-TetR-mCherry plasmids were grown overnight at 37°C in M9 minimal medium supplemented with 0.25% sodium citrate (M9 + citrate) and a mixture of trace ions (44). Gentamicin (30 μg/ml) and carbenicillin (200 μg/ml) were added to the medium whenever necessary. Cells were then transferred to fresh M9 + citrate medium at an optical density at 600 nm (OD_600_) of 0.01 and incubated at 30°C. After 3 h of cell growth, expression of fluorescent-tagged TetR proteins was induced by adding 0.05 mM IPTG. Cells were collected at an OD_600_ of 0.1 and deposited on an agarose pad (1% agarose in M9 + citrate medium) and promptly observed using a fluorescence microscope (Olympus BX50 equipped with a BX-FLA mercury light source and a 100× 1.43-numerical-aperture oil immersion objective). Phase-contrast and fluorescent images were collected using a Spot Insight QE camera and analyzed using Nucleus and MicrobeJ (45, 46). Unless noted otherwise, DNA and condensin localization was quantified from still pictures. Demographs were constructed using an in-house MATLAB script. Pixel intensities in each cell image were projected onto the major axis of the cell, binned, and normalized. Cells were binned according to their length, oriented to accentuate their asymmetry (negative coordinates assigned to foci in single-focus cells and to cell halves with two foci in three-focus cells), and the projected pixel intensity averaged as a function of the coordinate on the major axis over all cells in a given bin.

### Time-lapse imaging.

For time-lapse imaging, cells were grown as described above. Cells were deposited onto agarose pads (1% agarose in M9 + citrate medium) and snapshots were captured at 3- or 5-min intervals, as indicated in the figures, using a fluorescence microscope (Olympus IX53 inverted microscope equipped with Sola light engine). Images were captured using an ORCA-Fusion digital camera (C14440). Time-lapse image sequence was processed using FIJI (47) and an in-house MATLAB script.

### Marker frequency analysis.

Marker frequency analysis was done as previously described (28). Briefly, the genomic DNA was isolated using a GenElute bacterial genomic DNA kit (Sigma-Aldrich). The DNA was then subjected to deep sequencing, and relative abundancies of each DNA site were binned and fitted to a model of bidirectional replication using in-house MATLAB script. In this model, replication forks are assumed to advance by a constant number of base pairs for each micrometer of cell growth; hence the rate of the relative DNA replication is expressed in Mbp/μm. The model uses the following four adjustable parameters: the relative rates of clockwise and counterclockwise DNA replication and the cell lengths for the first and second rounds of *oriC* replication. These parameters were determined by fitting the model to the measured abundancies of genomic DNA fragments and the distribution of cell sizes.
