# Supplementary figures and images for: Alternating Dynamics of oriC, SMC, and MksBEF in Segregation of Pseudomonas aeruginosa Chromosome
Source: mSphere. 2020 Sep 9;5(5):e00238-20. doi: 10.1128/mSphere.00238-20 (PMC7485682; doi:10.1128/mSphere.00238-20)

A

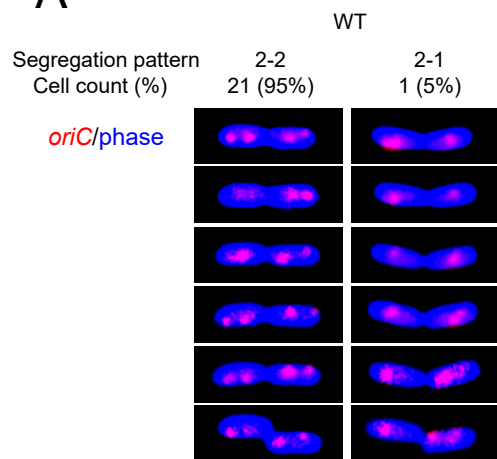

B

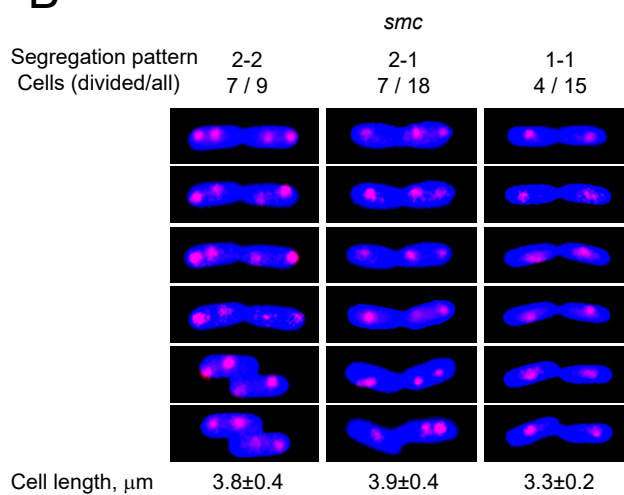

Supplement: FIG S1 [file mSphere.00238-20-sf001.pdf]

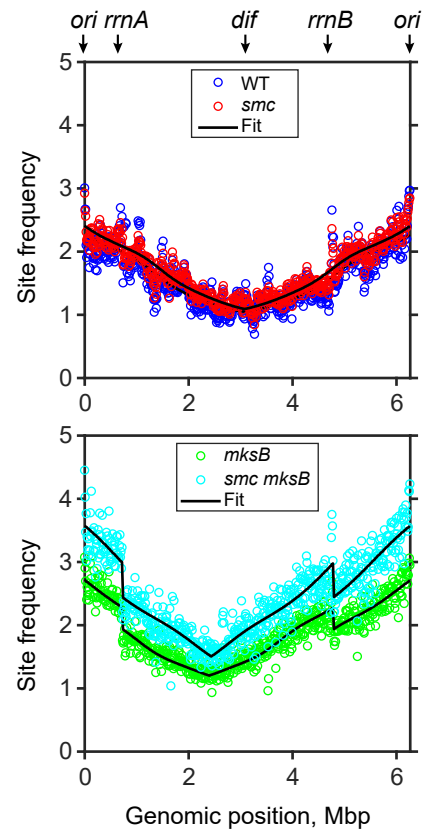

Supplement: FIG S2 [file mSphere.00238-20-sf002.pdf]

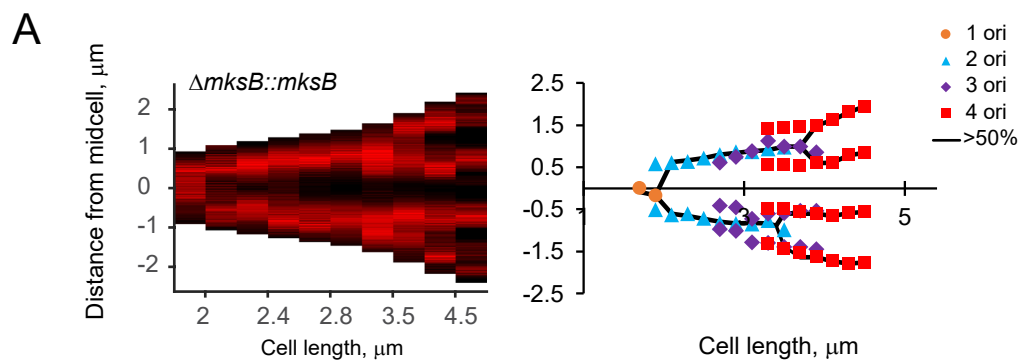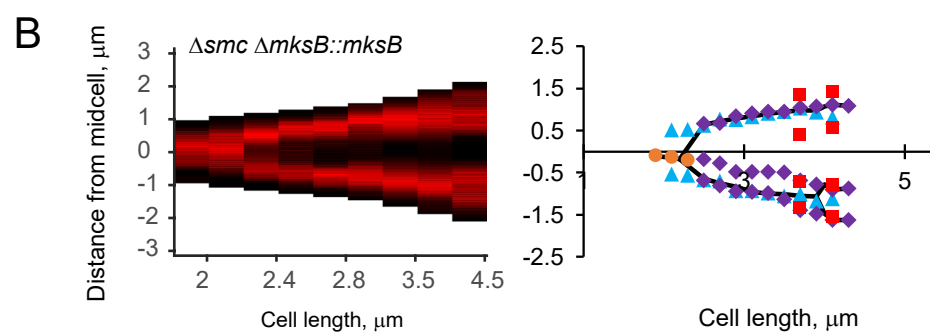

Supplement: FIG S3 [file mSphere.00238-20-sf003.pdf]

A

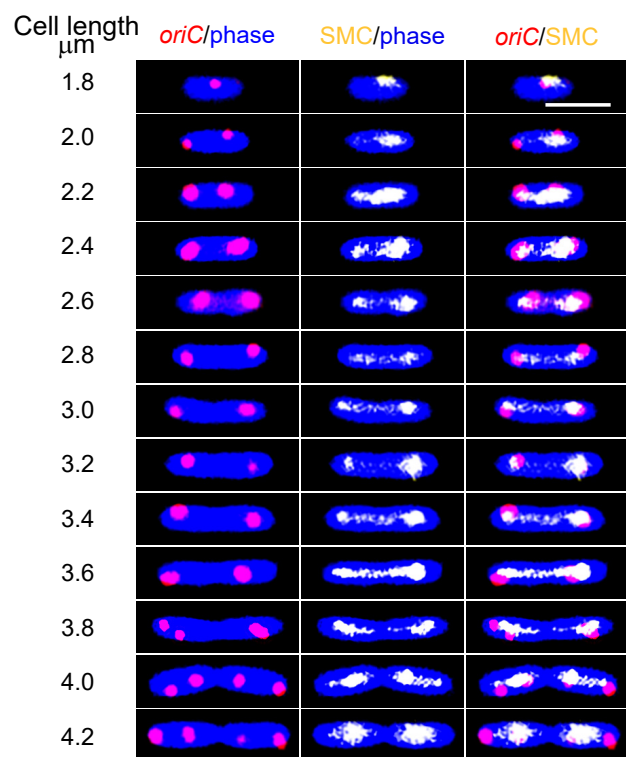

B

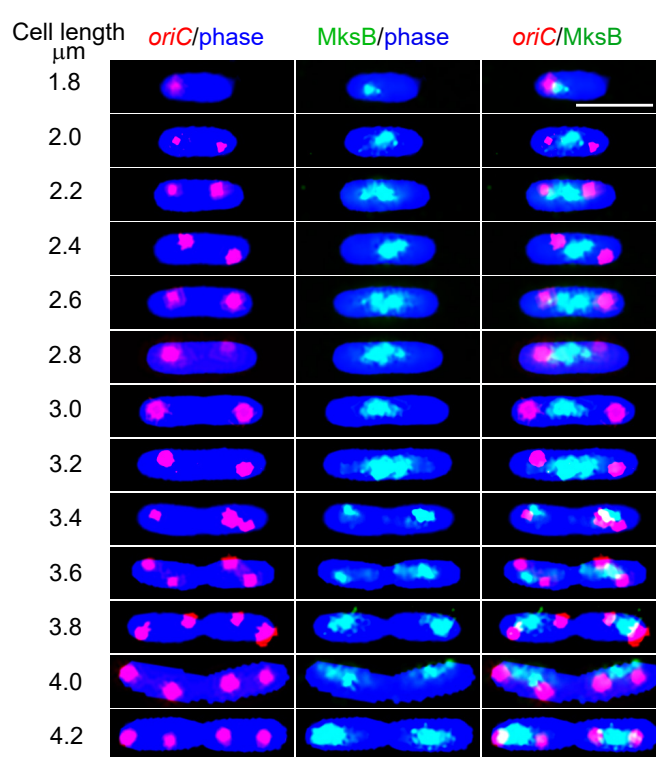

Supplement: FIG S4 [file mSphere.00238-20-sf004.pdf]

A

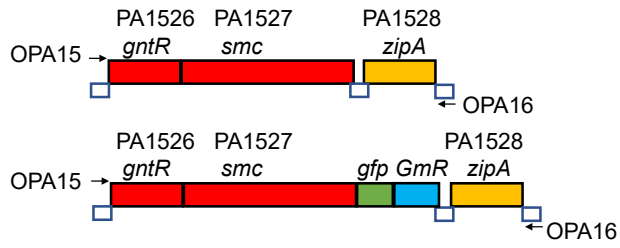

B

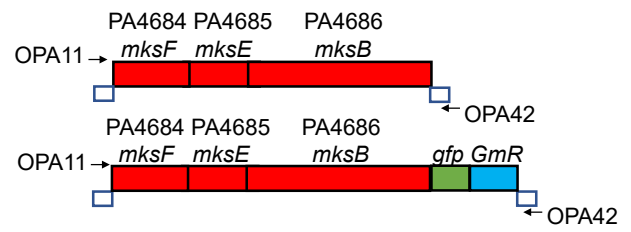

C

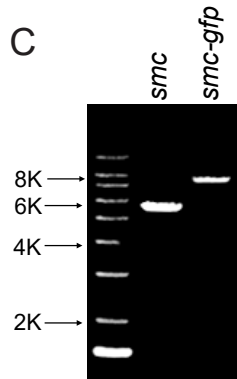

D

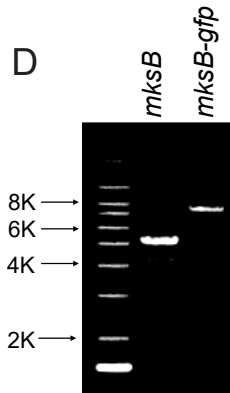

E

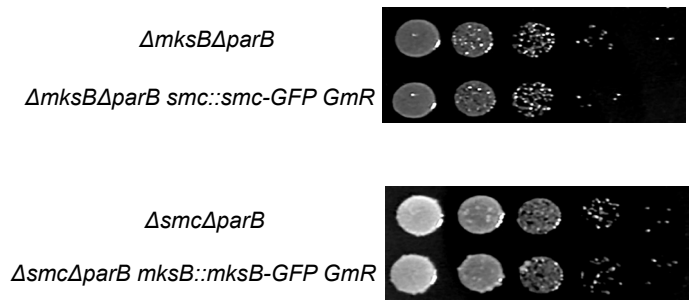

Supplement: FIG S5 [file mSphere.00238-20-sf005.pdf]
