# Supplementary material for: Alternating Dynamics of oriC, SMC, and MksBEF in Segregation of Pseudomonas aeruginosa Chromosome
Source: mSphere. 2020 Sep 9;5(5):e00238-20. doi: 10.1128/mSphere.00238-20 (PMC7485682; doi:10.1128/mSphere.00238-20)
Supplement: TABLE S1 [file mSphere.00238-20-st001.pdf]

Table S1. Strains used in this study.

Strain	Relevant genotype or description	Source or reference
DH5α	*supE44 ΔlacU169 hsdR17 recA1 endA1 gyrA96 thi-1 relA1*	Lab stock
SM10(λ pir)	*thi thr leu tonA lacY supE recA*::RP4-2-Tc::Mu Km λpir	Lab stock
PAO1-UW	*lacI*$^{q+}$ delta(*lacZ*)M15^{+} *tetA*$^{+}$ *tetR*$^{+}$	ATCC 47085
PAO1-DSM		(1)
OP107	PAO1 *Δsmc* ΔGm	(2)
OP109	PAO1 *ΔmksB* ΔGm	(2)
OP113	PAO1 *ΔmksB Δsmc* ΔGm	(2)
OP121	PAO1 *mksB::mksB-GFP* Gm FRT	This study
OP123	PAO1 *smc::smc-GFP* Gm FRT	This study
OP130	PAO1 *ΔsspB*	(2)
OP435	PAO1 *ΔparB Δsmc*	This study
OP441	PAO1 *ΔparB ΔmksB*	This study
OP475	PAO1 *ΔsspB Δsmc ΔparB*	This study
OP489	PAO1 *ΔsspB ΔmksB ΔparB*	This study
OP132	PAO1 *ΔsspB mksB::mksB*-DAS4 ΔGm	(2)
OP498	PAO1 *ΔsspB smc::smc*-DAS4 ΔGm	This study
OP501	PAO1 *ΔsspB ΔparB smc::smc*-DAS4 ΔGm	This study
OP508	PAO1 *ΔsspB ΔparB mksB::mksB*-DAS4 ΔGm	This study
OP505	PAO1 *ΔsspB ΔparB ΔmksB smc::smc*-DAS4 ΔGm	This study
OP440	PAO1 *ΔsspB ΔparB Δsmc mksB::mksB*-DAS4 ΔGm	This study
OP506	PAO1 *ΔsspB ΔparB smc::smc*-DAS4 *mksB::mksB*-DAS4 ΔGm	This study

OP579	PAO1 *smc::smc-mVenus* tetO-PA0069 + pPSV35Ap-TetR-mCherry	This study
OP385	PAO1 *mksB::mksB-gfp* tetO-PA0069 + pPSV35Ap-TetR-mCherry	This study
OP461	PAO1 tetO-PA0069 + pPSV35Ap-TetR-mCherry	This study
OP462	PAO1 *ΔmksB* tetO-PA0069 + pPSV35Ap-TetR-mCherry	This study
OP457	PAO1 *Δsmc* PA0069 + pPSV35Ap-TetR-mCherry	This study
OP465	PAO1 *Δsmc ΔmksB* tetO-PA0069 + pPSV35Ap-TetR-mCherry	This study
OP493	PAO1 *ΔmksB ΔparB Δsmc (merodeploid)*	This study
OP487	PAO1 *Δsmc ΔparB ΔmksB (merodeploid)*	This study
OP578	PAO1 Δ*mexGHID (merodiploid)*	This study
OP579	PAO1 *ΔmksB ΔparB smc::smc-GFP* Gm FRT	This study
OP580	PAO1 *Δsmc ΔparB mksB::mksB-GFP* Gm FRT	This study
OP581	PAO1 *ΔmksB::mksB* tetO-PA0069 + pPSV35Ap-TetR-mCherry	This study
OP582	PAO1 *Δsmc ΔmksB::mksB* tetO-PA0069 + pPSV35Ap-TetR-mCherry	This study
OP560 (PΔ6)	PAO1 *ΔmexAB-oprM ΔmexCD-oprJ ΔmexXY ΔmexEF-oprN ΔtriABC ΔmexJK*	This study
OP562 (PΔ6Pore)	PAO1 *ΔmexAB-oprM ΔmexCD-oprJ ΔmexXY ΔmexEF-oprN ΔtriABC ΔmexJK FhuA*	This study
OP570	PΔ6Pore *ΔparB*	This study
OP573	PΔ6Pore *ΔparB Δsmc*	This study
OP575	PΔ6Pore *ΔparB ΔmksB*	This study
OP583	PΔ6Pore *ΔmksB*	This study

OP584	PΔ6Pore *Δsmc*	This study
OP585	PΔ6Pore *ΔmksB Δsmc*	This study
BKB170	PAO1 *ΔmksB* tetO-PA0069 + pPSV35Ap-TetR-CFP	This study
BKB243	PAO1 *ΔmksB* tetO-PA0460 + pPSV35Ap-TetR-CFP	This study
BKB260	PAO1 *ΔmksB* tetO-PA0716 + pPSV35Ap-TetR-CFP	This study
BKB175	PAO1 *ΔmksB* tetO-PA0981 + pPSV35Ap-TetR-CFP	This study
BKB271	PAO1 *ΔmksB* tetO-PA1436 + pPSV35Ap-TetR-CFP	This study
BKB286	PAO1 *ΔmksB* tetO-PA1673 + pPSV35Ap-TetR-CFP	This study
BKB250	PAO1 *ΔmksB* tetO-PA1905 + pPSV35Ap-TetR-CFP	This study
BKB174	PAO1 *ΔmksB* tetO-PA2258 + pPSV35Ap-TetR-CFP	This study
BKB212	PAO1 *ΔmksB* tetO-PA2910 + pPSV35Ap-TetR-CFP	This study
BKB287	PAO1 *ΔmksB* tetO-PA3035 + pPSV35Ap-TetR-CFP	This study
BKB238	PAO1 *ΔmksB* tetO-PA3267 + pPSV35Ap-TetR-CFP	This study
BKB171	PAO1 *ΔmksB* tetO-PA3573 + pPSV35Ap-TetR-CFP	This study
BKB213	PAO1 *ΔmksB* tetO-PA4457 + pPSV35Ap-TetR-CFP	This study
BKB279	PAO1 *ΔmksB* tetO-PA5099 + pPSV35Ap-TetR-CFP	This study
BKB326	PAO1 *Δsmc* tetO-PA0069 + pPSV35Ap-TetR-mCherry	This study
BKB336	PAO1 *Δsmc* tetO-PA0460 + pPSV35Ap-TetR-mCherry	This study
BKB328	PAO1 *Δsmc* tetO-PA0716 + pPSV35Ap-TetR-mCherry	This study
BKB329	PAO1 *Δsmc* tetO-PA0981 + pPSV35Ap-TetR-mCherry	This study
BKB337	PAO1 *Δsmc* tetO-PA1436 + pPSV35Ap-TetR-mCherry	This study
BKB338	PAO1 *Δsmc* tetO-PA1905 + pPSV35Ap-TetR-mCherry	This study
BKB315	PAO1 *Δsmc* tetO-PA2258 + pPSV35Ap-TetR-mCherry	This study
BKB314	PAO1 *Δsmc* tetO-PA2910 + pPSV35Ap-TetR-mCherry	This study

BKB320	PAO1 *Δsmc* tetO-PA3267 + pPSV35Ap-TetR-mCherry	This study
BKB321	PAO1 *Δsmc* tetO-PA3573 + pPSV35Ap-TetR-mCherry	This study
BKB312	PAO1 *Δsmc* tetO-PA4457 + pPSV35Ap-TetR-mCherry	This study
BKB339	PAO1 *Δsmc* tetO-PA5099 + pPSV35Ap-TetR-mCherry	This study

Supplemental References

1. Klockgether J, Munder A, Neugebauer J, Davenport CF, Stanke F, Larbig KD, Heeb S, Schock U, Pohl TM, Wiehlmann L, Tummler B. 2010. Genome diversity of Pseudomonas aeruginosa PAO1 laboratory strains. J Bacteriol 192:1113-21.

2. Zhao H, Clevenger AL, Ritchey JW, Zgurskaya HI, Rybenkov VV. 2016. Pseudomonas aeruginosa Condensins Support Opposite Differentiation States. J Bacteriol 198:2936-2944.
