# Supplementary material for: Alternating Dynamics of oriC, SMC, and MksBEF in Segregation of Pseudomonas aeruginosa Chromosome
Source: mSphere. 2020 Sep 9;5(5):e00238-20. doi: 10.1128/mSphere.00238-20 (PMC7485682; doi:10.1128/mSphere.00238-20)
Supplement: TABLE S2 [file mSphere.00238-20-st002.pdf]

Table S2. Plasmids used in this study.

Plasmids	Description	Genomic location	Source or reference
pP30D-FRT-TetO-0069	Insertion of *tetO* repeats at 12h (0.16h)	PA0069	(1)
pP30D-FRT-TetO-0460	Insertion of *tetO* repeats at 1h (0.99h)	PA0460	(2)
pP30D-FRT-TetO-0716	Insertion of *tetO* repeats at 1.5h (1.51h)	PA0716	(2)
pP30D-FRT-TetO-0981	Insertion of *tetO* repeats at 2h (2.04h)	PA0981	(1)
pP30D-FRT-TetO-1436	Insertion of *tetO* repeats at 3h (2.99h)	PA1436	(2)
pP30D-FRT-TetO-1673	Insertion of *tetO* repeats at 3.5h (3.50)	PA1673	This study
pP30D-FRT-TetO-1905	Insertion of *tetO* repeats at 4h (3.98h)	PA1905	(2)
pP30D-FRT-TetO-2258	Insertion of *tetO* repeats at 5h (4.76h)	PA2258	(1)
pP30D-FRT-TetO-2910	Insertion of *tetO* repeats at 6h (5.75h)	PA2910	(2)
pP30D-FRT-TetO-3035	Insertion of *tetO* repeats at 6.5h (6.51h)	PA3035	(2)
pP30D-FRT-TetO-3267	Insertion of *tetO* repeats at 7h (6.99h)	PA3267	(2)
pP30D-FRT-TetO-3573	Insertion of *tetO* repeats at 8h (7.67h)	PA3573	(1)
pP30D-FRT-TetO-4457	Insertion of *tetO* repeats at 10h (9.6h)	PA4457	(2)
pP30D-FRT-TetO-5099	Insertion of *tetO* repeats at 11h (10.99h)	PA5099	(2)
pPSV35Ap-TetR-CFP	Expression of TetR-CFP		This study

pPSV35Ap-TetR-mCherry	Expression of TetR-mCherry	This study
pFLP2	Site-specific excision vector	(3)
pEXG2	Allele exchange vector, Gm^R, sucrose counterselection	(4)
pEXG2-*ΔsspB*	*sspB* deletion vector	(4)
pEXG2-*ΔparB*	*parB* deletion vector	This study
pEX18AP	Allele exchange vector, Amp^R, sucrose counterselection	(3)
pEX18Ap-*Δsmc*	*smc* deletion vector	(5)
pEX18Ap-*ΔmksB*	*mksB* deletion vector	(5)
pEX-*ΔmksB::mksB*	Reinsertion of *mksB* into endogenous locus	This study
pEX18AP-*ΔmexAB-oprM*	*mexAB-oprM* deletion vector	This study
pEXG2-*ΔmexCD-oprJ*	*mexCD-oprJ* deletion vector	This study
pEXG2-*ΔmexXY*	*mexXY* deletion vector	This study
pEXG2-*ΔmexEF-oprN*	*mexEF-oprN* deletion vector	This study
pEXG2-*ΔmexJK*	*mexJK* deletion vector	This study
pEXG2-*ΔtriABC*	*triABC* deletion vector	This study
pGK-LAC-*fhuA* ΔC/Δ4L	FhuA insertion vector	(6)
pEX18AP-*smc-gfp*	*smc-gfp* tagging vector	This study
pEX18AP-*mksB-gfp*	*mksB-gfp* tagging vector	This study
pBAD-*mVenus*	Source of *mVenus* gene	Addgene
pEX18AP-*smc-mVenus*	*smc-mVenus* tagging vector	This study
pEX18AP-*mksB*-DAS4	*mksB-DAS4* tagging vector	(5)
pEX18AP-*smc*-DAS4	*smc-DAS4* tagging vector	This study

pPSPK-*sspB*	IPTG inducible SspB expression plasmid	(4)

Supplemental References

1. Vallet-Gely I, Boccard F. 2013. Chromosomal organization and segregation in Pseudomonas aeruginosa. PLoS genetics 9:e1003492.

2. Bhowmik BK, Clevenger AL, Zhao H, Rybenkov VV. 2018. Segregation but Not Replication of the Pseudomonas aeruginosa Chromosome Terminates at Dif. mBio 9.

3. Hoang TT, Karkhoff-Schweizer RR, Kutchma AJ, Schweizer HP. 1998. A broad-host-range Flp-FRT recombination system for site-specific excision of chromosomally-located DNA sequences: application for isolation of unmarked Pseudomonas aeruginosa mutants. Gene 212:77-86.

4. Castang S, McManus HR, Turner KH, Dove SL. 2008. H-NS family members function coordinately in an opportunistic pathogen. Proc Natl Acad Sci U S A 105:18947-52.

5. Zhao H, Clevenger AL, Ritchey JW, Zgurskaya HI, Rybenkov VV. 2016. Pseudomonas aeruginosa Condensins Support Opposite Differentiation States. J Bacteriol 198:2936-2944.

6. Krishnamoorthy G, Wolloscheck D, Weeks JW, Croft C, Rybenkov VV, Zgurskaya HI. 2016. Breaking the Permeability Barrier of Escherichia coli by Controlled Hyperporination of the Outer Membrane. Antimicrob Agents Chemother 60:7372-7381.
